# A Bizarre Planthopper Nymph (Hemiptera: Fulgoroidea) from Mid-Cretaceous Kachin Amber

**DOI:** 10.3390/insects12040318

**Published:** 2021-04-02

**Authors:** Cihang Luo, Bo Wang, Edmund A. Jarzembowski

**Affiliations:** 1 State Key Laboratory of Palaeobiology and Stratigraphy, Nanjing Institute of Geology and Palaeontology and Center for Excellence in Life and Paleoenvironment, Chinese Academy of Sciences, 39 East Beijing Road, Nanjing 210008, China; bowang@nigpas.ac.cn (B.W.); jarzembowski2@live.co.uk (E.A.J.); 2 University of Chinese Academy of Sciences, Beijing 100049, China

**Keywords:** planthoppers, Kachin amber, nymph, *Spinonympha* gen. nov., *Spinonympha shcherbakovi* sp. nov., new genus, new species, Cretaceous

## Abstract

**Simple Summary:**

The fossil record of adult planthoppers is relatively rich, but the nymphs are rare and not well studied. Here, we describe a bizarre armoured planthopper nymph: *Spinonympha shcherbakovi* gen. et sp. nov. from mid-Cretaceous Kachin amber. The new genus and species is characterized by its large size, armoured body, extremely long rostrum, and leg structure. The fossil nymph cannot be attributed to any known planthopper family, but can be excluded from many families due to its large size and leg structure. The armoured body was probably developed for defence, and the extremely long rostrum indicates that, in the past, planthopper feeding on trees with thick and rough bark was more widespread than today. The new find reveals a new armoured morphotype previously unknown in planthopper nymphs.

**Abstract:**

The fossil record of adult planthoppers is comparatively rich, but nymphs are rare and not well studied. Here, we describe a bizarre armoured planthopper nymph, *Spinonympha shcherbakovi* gen. et sp. nov., in mid-Cretaceous Kachin amber. The new genus is characterized by its large size, body armed with spines and tubercles, extremely long rostrum reaching well beyond the apex of the abdomen; profemur and mesofemur subcylindrical, covered with setae; protibia and mesotibia subquadrangular, densely covered with setae; protarsus and mesotarsus with two segments, tarsomere II longer and wider than I; metatrochanter swollen, metafemur subcylindrical, covered with short setae; metatibia subquadrangular, densely covered with short setae, without lateral spine and pectens without setae; metatarsus with three segments, and metatarsomere III extremely small. The fossil nymph cannot be attributed to any known planthopper family, but can be excluded from many families due to its large size and leg structure. The armoured body was probably developed for defence, and the extremely long rostrum indicates that, in the past, feeding on trees with thick and rough bark was more widespread than today. These features indicate that the new specimen represents a new armoured morphotype of planthopper nymph from the fossil record.

## 1. Introduction

The order Hemiptera is the fifth largest order among living insects [1,2], with more than three hundred extant and extinct families, inhabiting almost all terrestrial and some marine habitats [2].

Although the fossil record of planthoppers is relatively abundant (more than 350 species in 25 families [3,4,5,6]), and studies on the nymphs of extant Fulgoroidea having advanced considerably in the last few decades [7], fossil planthopper nymphs have received comparatively little attention [4,8,9]. The oldest fossil nymph attributed to Fulgoroidea is *Knezouria unicus* Jell, 1993, reported from the Late Triassic (Carnian, 237–227 million years ago) of the Ipswich Basin, Queensland, Australia [10], but several critical characters are unclear, and the placement of this taxon is inconclusive [8]. Szwedo [8] established a new extinct family Neazoniidae Szwedo, 2007 based on three nymphs (*Neazonia tripleta* Szwedo, 2007, *N. immatura* Szwedo, 2007, and *N. imprinta* Szwedo, 2007) from Lower Cretaceous Lebanese amber (approximately 135 million years ago). Later, another new genus of this family (based on *Akmazeina santonorum* Szwedo, 2007) was discovered in Lower Cretaceous French amber (approximately 100 million years ago) [9]. Shcherbakov [11] reported two genera of nymphs belonging to Perforissidae Shcherbakov, 2007: *Perforissus muiri* Shcherbakov, 2007 (first instar) and *Cretargus shcherbakovi* Shcherbakov, 2007 (first instar) [9,11]. Emeljanov and Shcherbakov [12] described a new Eocene (56–34 million years ago) genus and species of Dictyopharidae, *Alicodoxa rasnitsyni* Emeljanov et Shcherbakov, 2011 (Dictyopharinae: Orthopagini) from the Rovno (NW Ukrainian) and Baltic ambers, based on nymphs, and very recently another new genus and species, *Bathymyza longirostris* Emeljanov and Shcherbakov, 2020 (Dictyopharidae: Dictyopharinae: Orthopagini?) was described based on an early instar nymph from Bitterfeld amber (Saxony-Anhalt in Germany) [13]. Emeljanov and Shcherbakov [14] also established a new family Dorytocidae with the longest proboscis among Mesozoic Fulgoroidea, based on the nymphs (*Dorytocus ornithorhynchus* Emeljanov and Shcherbakov, 2018) of three different instars from mid-Cretaceous Kachin amber (approximately 100 million years ago). However, the adults of these nymphs remain unknown.

Herein we describe a new and bizarre armoured planthopper fossil nymph, *Spinonympha shcherbakovi* gen. et sp. nov., from mid-Cretaceous Kachin (Burmese) amber from northern Myanmar.

## 2. Materials and Methods

The studied specimen comes from the Cretaceous deposits of the Hukawng Valley in Myanmar, from a former amber mine located near Danai (Tanai) Town (26°21′33.41″ N, 96°43′11.88″ E; palaeolatitude 5.0 ± 4.7° S) [15,16]. Over the last 100 years, and particularly in the past two decades, Kachin amber has received worldwide scientific interest; more than 600 families of invertebrates, vertebrates, protists, plants, and fungi have been reported [17,18,19,20]. The Burma Terrane, in which the amber occurs, was part of a Trans-Tethyan island arc at a near-equatorial more-southern latitude at about 95  million years ago, suggesting island endemism for the Kachin amber biota [15]. The Kachin amber is still giving us new insights into the very important period of formation of modern faunistic complexes during the mid-Cretaceous biotic re-organization [21] and provides ideal material for studying the Cretaceous Terrestrial Revolution, which is marked by the radiation of angiosperms, social insects, and mammals [22,23,24,25]. Radiometric U-Pb zircon dating of the volcaniclastic matrix of the amber constrained a refined age of 98.79 ± 0.62 million years ago (earliest Cenomanian) [26].

The specimen studied in the course of this work is deposited in Nanjing Institute of Geology and Palaeontology, Chinese Academy of Sciences, Nanjing. To avoid any confusion and misunderstanding, all authors declare that the fossil reported in this study was not involved in armed conflict and ethnic strife in Myanmar. This specimen is deposited permanently in a publicly owned collection in a national museum, in full compliance with the International Code of Zoological Nomenclature and Statement of the International Palaeoentomological Society [27,28].

The photographs were taken with a Zeiss Stereo Discovery V16 microscope system in the Nanjing Institute of Geology and Palaeontology, Chinese Academy of Sciences, and measurements were taken using Zen software. Photomicrographic composites of 50 individual focal planes were digitally stacked as obtained using the software HeliconFocus 6.7.1 for a better illustration of 3D structures. Photographs were adjusted using Adobe Lightroom Classic and line drawings were prepared using CorelDraw 2019 graphic software.

The morphological terminology used in this study mostly follows Luo et al. [5].

## 3. Systematic Palaeontology

Hemiptera Linnaeus, 1758

Fulgoromorpha Evans, 1946

Fulgoroidea Latreille, 1807

Family incertae sedis

**Figure 1:**
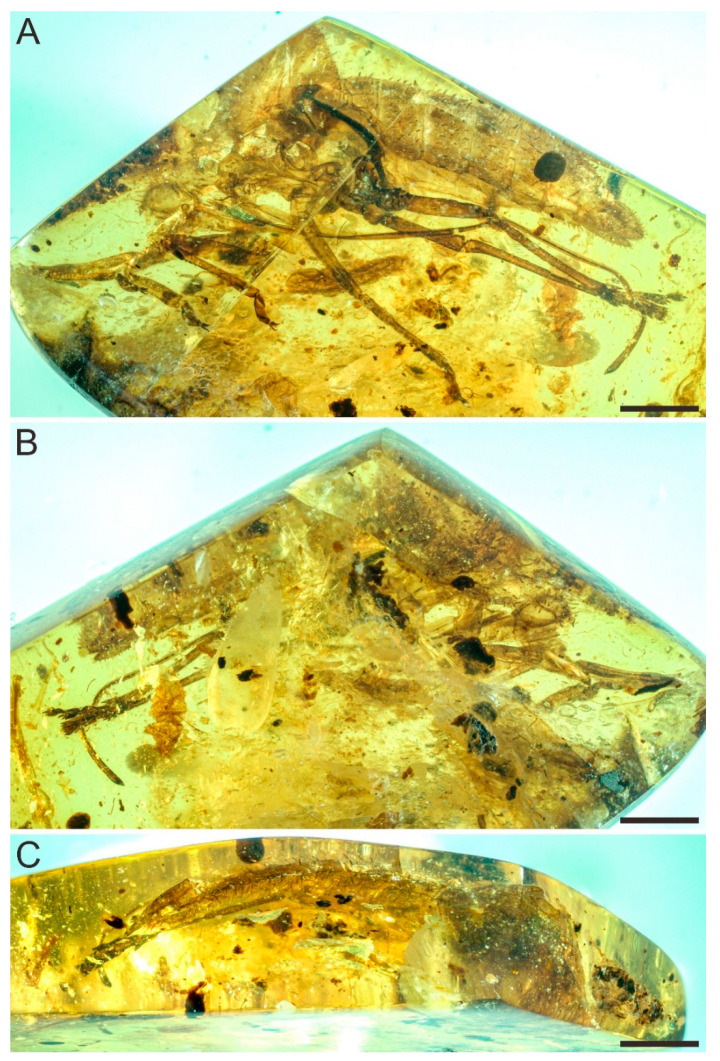
Holotype of *Spinonympha shcherbakovi* gen. et sp. nov. (NIGP174758). (**A**) Left lateral view. (**B**) Right lateral view. (**C**) Dorsal view. Scale bars = 2 mm.

**Figure 2:**
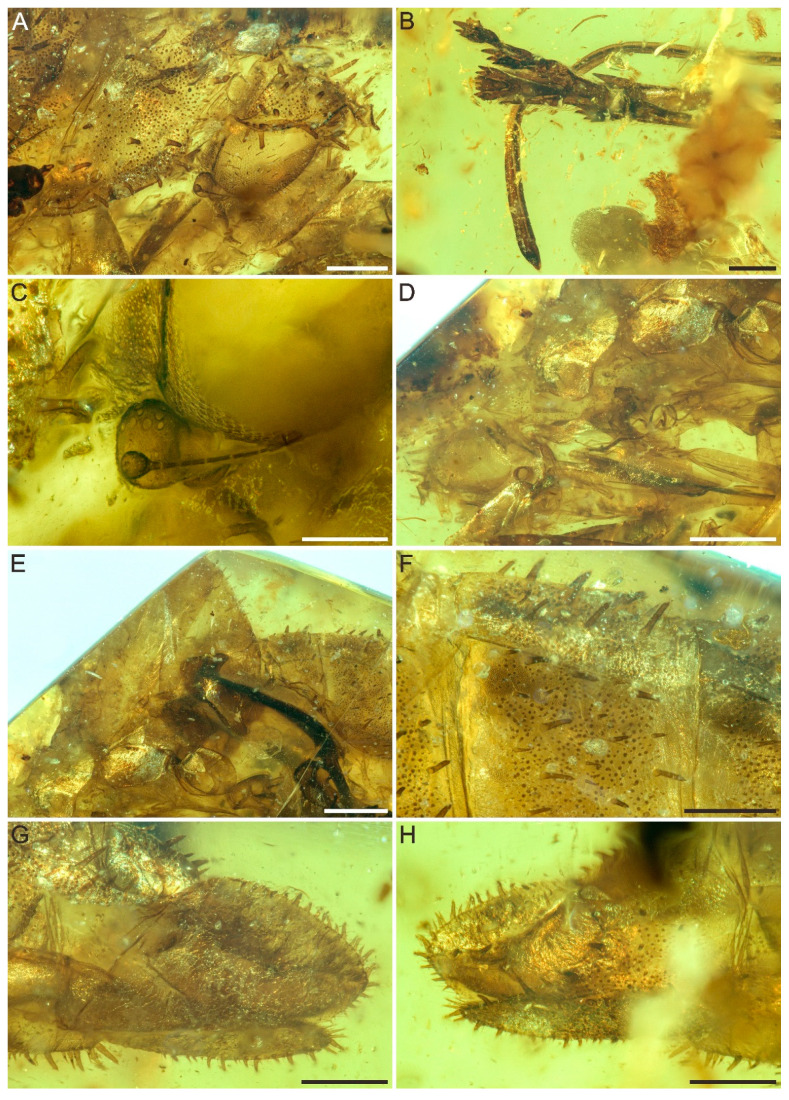
Detailed photographs of the head, pronotum, mesonotum, and metanotum of *Spinonympha shcherbakovi* gen. et sp. nov. (**A**) Head and pronotum in right lateral view. (**B**) Apex of rostrum in right lateral view. (**C**) Antenna in right lateral view. (**D**) Pronotum and mesonotum in left lateral view. (**E**) Metanotum in left lateral view. (**F**) Spines and tubercles of abdomen in left lateral view. (**G**) Pygofer in left lateral view. (**H**) Pygofer in right lateral view. Scale bars for (**D**,**E**) = 1.0 mm; (**A**,**B**,**F**–**H**) = 0.5 mm; (**C**) = 0.2 mm.

**Figure 3:**
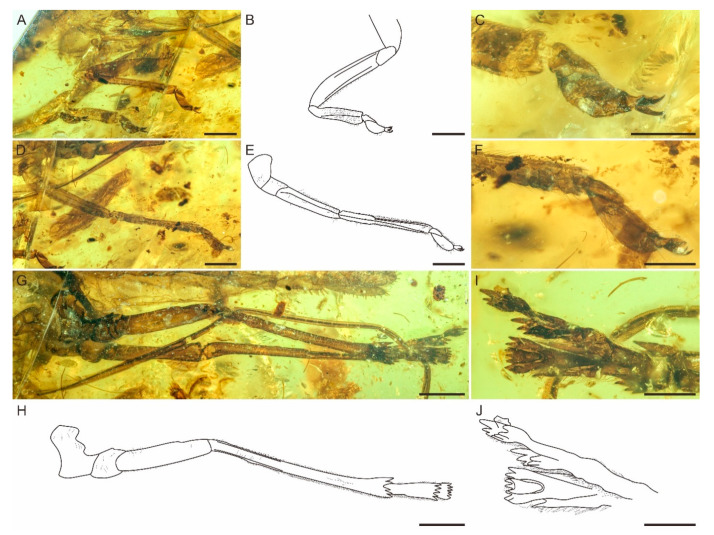
Detailed photographs and line drawing of legs of *Spinonympha shcherbakovi* gen. et sp. nov. (**A**) Left foreleg in left lateral view. (**B**) Line drawing of left foreleg in left lateral view. (**C**) Protarsus. (**D**) Left midleg in left lateral view. (**E**) Line drawing of left midleg in left lateral view. (**F**) Mesotarsus. (**G**) Hindleg in left lateral view. (**H**) Line drawing of left hindleg in left lateral view. (**I**) Mesotarsus. (**J**) Line drawing of mesotarsus. Scale bars for (**A**,**B**,**D**,**E**,**G**,**H**) = 1.0 mm; (**C**,**F**,**I**,**J**) = 0.5 mm.

Genus ***Spinonympha* Luo, Wang et Jarzembowski gen. nov.** (Figure 1, Figure 2 and Figure 3).

urn:lsid:zoobank.org:act:B0CBFCC7-059A-49C5-8C3F-8F4DC57BD831

Type species. *Spinonympha shcherbakovi* sp. nov.; by present designation.

Etymology. The generic name is derived from the combination of two words from the Latin language: “Spino-” meaning “spiny”, and “nympha” meaning “nymph”, referring to the spiny body of the nymph. Gender: Feminine.

Included species. Type species only.

Diagnosis. Large nymph, body covered by numerous spines and tubercles (armoured); rostrum extremely long, reaching well beyond apex of abdomen; profemur subcylindrical, carinate, covered with short setae; protibia subquadrangular, carinate, densely covered with short setae; protarsus with two segments, protarsomere II longer and wider than protarsomere I; mesofemur subcylindrical, carinate, covered with short setae; mesotibia subquadrangular, carinate, densely covered with short setae, thinnest near middle; mesotarsus with two segments, mesotarsomere II longer and wider than mesotarsomere I; metatrochanter swollen, metafemur subcylindrical, carinate, covered with short setae; metatibia subquadrangular, carinate, densely covered with short setae, without lateral spine, pectens without setae; metatarsus with three segments, metatarsomere III extremely small; metatibio-tarsal formula 5:5:7.

Age and occurrence. Mid-Cretaceous (earliest Cenomanian, approximately 100 million years ago); amber from Kachin State, northern Myanmar.

***Spinonympha shcherbakovi* Luo, Wang et Jarzembowski sp. nov.** (Figure 1, Figure 2 and Figure 3).

urn:lsid:zoobank.org:act:32400541-FFBC-4373-B325-2BB8006AF8F5

Etymology. The specific name is dedicated to Prof. Dmitry E. Shcherbakov, an eminent researcher on fossil insects.

Material. Holotype, female nymph. Specimen No. NIGP174758, deposited in Nanjing Institute of Geology and Palaeontology, Chinese Academy of Sciences, Nanjing. Cabochon, 22 × 12 × 4 mm, weight 1.0 g. Holotype inclusion incomplete: Flattened exuvium, so almost only visible in lateral view.

Locality and horizon. Kachin amber, near Tanai Village in the Hukawng Valley of northern Myanmar, earliest Cenomanian (mid-Cretaceous).

Diagnosis. As for genus.

Description. Large female nymph, total length of body 14.5 mm, body covered by numerous spines and tubercles (Figure 1). Vertex with round anterior margin, slightly extending and covering compound eyes, about 0.94 mm long (Figure 2A). Frons not visible. Clypeus at least 2.0 mm long. Rostrum extremely long, reaching well beyond apex of abdomen, slightly thickened near apex, 12.5 mm long in total (Figure 2B). Compound eyes with several setae, 0.85 mm long and 0.68 mm wide in lateral view. Median ocellus and lateral ocelli not visible. Antennal foveae elevated, ring-like; scape very short, ring-like, about 0.03 mm long; pedicel mushroom-like, with more than 30 distinct round sensory plaque organs, 0.32 mm long and 0.21 mm wide at widest part; flagellum very swollen in basal part, then whip-like, about 0.43 mm long (Figure 2C). Pronotum 0.64 mm long in lateral view. Mesonotum 1.1 mm long in lateral view (Figure 2D). Metanotum large, 3.95 mm long in lateral view, with straight anterior margin and round posterior margin (Figure 2E).

Foreleg (Figure 3A–C) with procoxa strong, cylindrical, slightly thinner towards apex, 0.98 mm long and 0.46 mm wide; protrochanter subcylindrical, carinate, widening towards apex, 0.59 mm long and 0.41 mm wide at widest point; profemur subcylindrical, carinate, covered with short setae, slightly thinner towards apex, 2.60 mm long and 0.46 mm wide; protibia subquadrangular, carinate, densely covered with short setae, slightly wider towards apex, 1.73 mm long and 0.41 mm wide at broadest point; protarsus with two segments: Protarsomere I subtriangular, margin covered with short setae, 0.36 mm long and 0.21 mm wide, protarsomere II longer and wider than protarsomere I, subcylindrical, carinate, covered with short setae, widest at middle, 0.61 mm long and 0.23 mm wide; tarsal claws and arolium developed: tarsal claws narrow, 0.27 mm long, arolium pad-like, with rounded apical margin, 0.17 mm long and 0.10 mm wide. Midleg (Figure 3D–F) with mesocoxa strong, cylindrical, thinner towards apex, 0.84 mm long and 0.69 mm wide; mesotrochanter subcylindrical, almost keeping same width towards apex, 0.62 mm long and 0.48 mm wide; mesofemur subcylindrical, carinate, covered with short setae, slightly thinner towards apex, 2.11 mm long and 0.49 mm wide at widest point; mesotibia subquadrangular, carinate, densely covered with short setae, thinnest nearmiddle, 2.93 mm long and 0.27 mm wide at widest point; mesotarsus with two segments: Mesotarsomere I subtriangular, margin covered with short setae, 0.39 mm long and 0.19 mm wide, mesotarsomere II longer and wider than mesotarsomere I, subcylindrical, carinate, covered with short setae, widest at middle, 0.79 mm long and 0.25 mm wide; tarsal claws and arolium developed: Tarsal claws narrow, 0.28 mm long, arolium pad-like, with rounded apical margin, 0.18 mm long and 0.11 mm wide. Hindleg (Figure 3G–J) with metacoxa stout, cylindrical, thinner towards apex, 1.06 mm long and 1.16 mm wide; metatrochanter subcylindrical, swollen, wider than metafemur, slightly wider towards apex, 0.53 mm long and 0.57 mm wide; metafemur subcylindrical, carinate, covered with short setae, almost keeping same width towards apex, 2.23 mm long and 0.45 mm wide; metatibia subquadrangular, carinate, densely covered with short setae, without lateral spine, thinnest near middle, with five apical teeth, 3.91 mm long and 0.23 mm wide; metatarsus with three segments: Metatarsomere I subcylindrical, widening towards apex, margin densely covered with short setae, with five apical teeth, 1.15 mm long and 0.35 mm wide, metatarsomere II subtriangular, covered with short setae, widening towards apex, with seven apical teeth, 0.90 mm long and 0.36 mm wide; metatarsomere III extremely small, subtriangular, widening towards apex, 0.31 mm long and 0.15 mm wide; tarsal claws small, arolium indistinct (Figure 3I,J). Metatibio-tarsal formula 5:5:7.

Abdomen with pygofer 9.61 mm long and 2.11 high in lateral view, 9-segmented, dorsal surface covered by numerous spines and tubercles (Figure 2F). Segment I mostly shielded by metanotum, approximately 1.05 mm long; segment II 0.90 mm long; segment III 1.07 mm long; segment IV 1.24 mm long; segment V 1.61 mm long; segment VI 0.98 mm long; segment VII 0.86 mm long; segment VIII approximately 0.46 mm long. Pygofer with round apex, 1.44 mm long, ovipositor visible in lateral view, slightly curved, apex acutely rounded, 1.32 mm long (Figure 2G,H).

## 4. Discussion

*Spinonympha shcherbakovi* Luo, Wang et Jarzembowski gen. et sp. nov. can be assigned to Hemiptera according to its piercing-sucking mouthparts, and it can be assigned to Fulgoromorpha due to its antenna positioned below the compound eyes and metatibio-tarsal pecten, but it cannot be attributed to Coleoscytoidea and Surijokocixioidea, and should be placed in Fuloroidea because the former two superfamilies went extinct after the Triassic. However, *S. shcherbakovi* cannot be assigned to any known family because the phylogenetic relationships of the Fulgoroidea are mainly based on characters of the adults and its relatively poor preservation (flattened exuvium), but it can be provisionally excluded from many planthopper families due to its large size (nearly 15 mm), e.g., Achilidae Stål, 1866, Cixiidae Spinola, 1839, Delphacidae Leach, 1815, Derbidae Spinola, 1839, Dictyopharidae Spinola, 1839, Issidae Spinola, 1839, Jubisentidae Zhang, Ren et Yao, 2019, Kinnaridae Muir, 1925, Neazoniidae Szwedo, 2007, Nogodinidae Melichar, 1898, Perforissidae Shcherbakov, 2007, Tropiduchidae Stål, 1866, and Yetkhatidae Song, Szwedo and Bourgoin, 2019.

The most striking morphological feature of the fossil is its body covered by numerous spines and tubercles (i.e., armour). Such structure is common among some insect larvae from mid-Cretaceous Kachin amber [29,30,31,32], but very rare in extant and extinct planthoppers. By the Early Cretaceous, many new predaceous arthropods (including some spiders, lacewing larvae, and ants) and vertebrates (including lizards, birds, and mammals) had appeared [33,34,35], so *S. shcherbakovi* probably used these spines and tubercles to protect itself from predators. However, we cannot completely exclude the possibility that these spines and tubercles were developed for mimicry of moss [36].

Sensory pits that are typically present on nymphs of planthoppers are not clearly preserved on *S. shcherbakovi*. A sensory pit is defined as “a small hole with horizontal seta directed inwards and diverging from its border; the length of the seta is not greater than diameter of the hole” [7,37]. Sensory pits are characteristic of Fulgoroidea nymphs in all families except in Tettigometridae and Hypochtonellidae [8], but they are very rare in adults [7]. They usually present on the head and thorax [9], and can also be found on the abdomen (e.g., [12,13,14]). Sensory pits present in all other fossil planthopper nymphs [8,9,10,11,12,13,14], so the absence of pits on *S. shcherbakovi* is probably because of poorly preservation.

Another interesting characteristic is the extremely long rostrum reaching beyond the apex of the abdomen, but this feature is not rare among Mesozoic planthoppers. A similar long rostrum can be found in Dictyopharidae [13], Dorytocidae [14], Fulgoridiidae [38,39], Mimarachnidae [40,41,42], and Neazoniidae [8,9], as well as in the poorly known genus *Knezouria* Jell, 1993 from the Upper Triassic of Australia, probably representing the suborder Fulgoromorpha, but with superfamily placement unknown [8], indicating that, in the past, planthopper feeding on trees with thick and rough bark might have been more widespread than today [13,14,40,43], and implying gymnosperms usually with thicker bark than angiosperms. It could represent a plesiomorphic character among basal or intermediate fulgoroid taxa, or the result of convergent evolution [8].

The legs of the new specimen do not differ significantly from the legs of various fulgoroid nymphs, but there are still some differences. *Spinonympha shcherbakovi* has a strong coxa, which is not so long and robust as in Dictyopharidae [44], Dorytocidae [13,14], Jubisentidae [45,46], and Mimarachnidae [42]. The profemur and mesofemur are subcylindrical, and protibia and mesotibia subquadrangular in *S. shcherbakovi* (versus protibia and mesotibia flattened and foliaceous in fossil Dorytocidae nymphs [14]; profemur and protibia, mesofemur and mesotibia dilated and foliaceous in Jubisentidae [45,46]). The protarsus and mesotarsus of the new species have only two segments (versus protarsus and mesotarsus usually with three segments in Dictyopharidae [44], Jubisentidae [45,46], Lalacidae [47], Perforissidae [11,48,49,50,51], and Tropiduchidae [52,53,54]). The metatibia is usually with lateral spines in Achilidae [55], Dictyopharidae [44], Katlasidae [5], Nogodinidae [56,57,58], Ricaniidae [59], Tropiduchidae [52,53,54], Yetkhatidae [60], and sometimes in Lalacidae [47], but our new genus does not have any lateral spines on its metatibia. The hind tibial pecten is usually setigerous in Jubisentidae [45,46], Lalacidae [47], and Perforissidae [11,48,49,50,51], and this characteristic is also absent in *S. shcherbakovi*. A hindleg with swollen metatrochanter and extremely small metatarsomere III are potential autapomorphies.

In summary, *S. shcherbakovi* cannot be attributed to any known planthopper family, but it would be arbitrary to establish a new family for it at present. The placement of *S. shcherbakovi* within Fulgoroidea depends on the discovery of additional specimens. Nevertheless, it represents a new morphotype of planthopper nymph not seen before in the recent and fossil record.

## 5. Conclusions

An armoured planthopper nymph *Spinonympha shcherbakovi* gen. et sp. nov. is described from mid-Cretaceous Kachin amber. The new genus and species is characterized by its large size, armoured body, extremely long rostrum, and leg structure. The function of the spines and tubercles on the body is probably defence against predators. The new finding enhances our knowledge of the taxonomic diversity and morphological disparity of planthopper nymphs.

## Data Availability

No new data were created or analysed in this study. Data sharing is not applicable to this article.
